# Genetic Architecture Promotes the Evolution and Maintenance of Cooperation

**DOI:** 10.1371/journal.pcbi.1003339

**Published:** 2013-11-21

**Authors:** Antoine Frénoy, François Taddei, Dusan Misevic

**Affiliations:** INSERM U1001, Université Paris Descartes, Sorbonne Paris Cité, Faculté de Médecine Paris Descartes, Paris, France; University of Texas at Austin, United States of America

## Abstract

When cooperation has a direct cost and an indirect benefit, a selfish behavior is more likely to be selected for than an altruistic one. Kin and group selection do provide evolutionary explanations for the stability of cooperation in nature, but we still lack the full understanding of the genomic mechanisms that can prevent cheater invasion. In our study we used Aevol, an agent-based, *in silico* genomic platform to evolve populations of digital organisms that compete, reproduce, and cooperate by secreting a public good for tens of thousands of generations. We found that cooperating individuals may share a phenotype, defined as the amount of public good produced, but have very different abilities to resist cheater invasion. To understand the underlying genetic differences between cooperator types, we performed bio-inspired genomics analyses of our digital organisms by recording and comparing the locations of metabolic and secretion genes, as well as the relevant promoters and terminators. Association between metabolic and secretion genes (promoter sharing, overlap via frame shift or sense-antisense encoding) was characteristic for populations with robust cooperation and was more likely to evolve when secretion was costly. In mutational analysis experiments, we demonstrated the potential evolutionary consequences of the genetic association by performing a large number of mutations and measuring their phenotypic and fitness effects. The non-cooperating mutants arising from the individuals with genetic association were more likely to have metabolic deleterious mutations that eventually lead to selection eliminating such mutants from the population due to the accompanying fitness decrease. Effectively, cooperation evolved to be protected and robust to mutations through entangled genetic architecture. Our results confirm the importance of second-order selection on evolutionary outcomes, uncover an important genetic mechanism for the evolution and maintenance of cooperation, and suggest promising methods for preventing gene loss in synthetically engineered organisms.

## Author Summary

Cooperation is a much studied and debated phenomena in the microbial world marked by a key question: Given the survival of the fittest evolutionary paradigm, why do individuals act in seemingly altruistic ways, paying a cost to help others? Kin selection and group selection, together with mathematical tools from areas such as economics and game theory, have provided some answers. However, they largely ignored the underlying genetic and genomic mechanisms that drive the evolution of cooperation. In this study, we show that the architecture of the genomes has a major role in shaping the fate of cooperating populations. Specifically, we use an *in silico* evolution platform and discover that genes for cooperative traits are “hiding” behind metabolic ones by overlapping their sequences or sharing operons. In conditions where cheaters may outcompete the cooperators, this entangled architecture evolves spontaneously and effectively protects cooperation from invasion by cheater mutants. We describe a novel genetic mechanism for the evolution and maintenance of cooperation and, by taking into account the second order selection pressures on the genomes, highlight the need for going beyond simple game theory models in its study.

## Introduction

The evolution of cooperation in microbial populations is a fascinating, rich and controversial evolutionary problem [1]–[6]. The theoretical understanding of cooperation has been gradually advancing for decades, and recently those insights have also been applied to practical, medical problems, such as the treatment of infections triggered by cooperating, pathogenic bacteria [7], [8]. Most evolutionary explanations of cooperation rely on kin selection and group selection theories and are constantly being improved and refined by a host of mathematical tools [9], [10]. Among them, the game theory and meta-population models have proved to be especially useful in the analysis of long term versus short term, as well as the individual versus population benefit of cooperation [11]–[14]. However, those methods tell us practically nothing about the evolutionary pressure on the structure of genomes that encode the cooperative traits. They typically do not distinguish between genotypes and phenotypes and consider only a finite set of possible behaviors (often only two: cooperate or not) with a constant extrinsic probability of switching between them. Although some recent papers do go further than evolving classical binary behavior by considering more complex stochastic strategies that take into account past interactions [15], they also remain “one locus = one parameter” models, unable to consider genetic architecture of cooperation genes. Several experimental studies have shown the need to go beyond these limitations to understand cooperation in microbial systems. Specifically, Foster *et al.* demonstrated that the pleiotropic effect of a *Dictyostelium discoideum* gene involved in a cooperative behavior (differentiation into prestalk cells) causes the mutations inducing cheating behavior to be associated with a direct fitness cost to the individual [16]. Similarly, cheating mutations induce a cost in *Pseudomonas aeruginosa* because of co-regulation of public and “private” goods via the same quorum-sensing mechanism [17].

We postulate that genomic architecture of metabolic and secretion genes – achieved by sense-antisense coding or frameshifts – can provide a mechanism for the evolution and maintenance of cooperation that is similar but more basic than ones relying on genetic pleiotropy or co-regulation. Here we investigate how two specific types of genomic architecture of cooperation genes may affect the evolutionary fate of cooperation itself. The first type relies on the concept of operons, already well described and investigated in the context of co-regulation or co-transfer of genes in the same operon [18], [19]. We specifically consider metabolic and secretion genes that have the same promoter and terminator sequence, thus sharing an operon. The second architecture type is the overlap, base-pair sharing between metabolic and secretion genes due to being in different reading frames or on different DNA strands. Although more rare in bacterial context, gene overlaps may be caused by the strong constraints on maximum genome size and have similar evolutionary explanations and properties as operons [20]. We describe and quantify the role of both these genetic architecture types and show that physical association of cooperation and metabolic genes, via operon and overlap, introduces an evolutionary constraint, pleiotropy in the broad sense, which prevents non-cooperating, cheater individuals from prospering and protects cooperation. Even though the same DNA coding for multiple proteins can, in a broad sense, be viewed as pleiotropy at the sequence level, as far as we know, its importance has never been described in the context of cooperation.

In all our experiments we use the Aevol platform [21], [22], an *in silico* experimental evolution system. While similar to existing individual-based, genetic-algorithm simulations, Aevol embodies a number of features inspired by microbial genetics that make it especially well suited for our study. For example, the phenotype of an Aevol digital organism is a continuous function comprised of a potentially unlimited number of biological processes and their performance level, which in turn allows for a continuous cooperating phenotype instead of the classical binary one. When it evolves, the cooperation among individuals is based on a public good molecule that diffuses and degrades in the environment. Individuals live in a spatially structured world, suitable for the evolution of cooperation [23], [24] and more similar to natural microbial populations than classical meta-population models. The public good is costly to secrete but may benefit any neighboring organisms. Both indirectly selected secretion genes and metabolic genes contributing to fitness directly are encoded in the double-stranded genomes strings of zeros and ones. A set of rules for transcription, translation and protein synthesis governs the complex genotype to phenotype to fitness mapping. Phenotypically similar or even identical individuals can have different genotypes, thus also having different evolvability, robustness, and evolutionary fate [25]. All these properties of Aevol set the stage for evolutionary experiments where genetic architecture constraints of cooperation can be both observed and described. We first demonstrate the existence of differences in the resistance to cheater invasion among several phenotypically equivalent populations. We then correlate the maintenance of cooperation genes with the abundance of promoter sharing or overlapping between metabolic and secretion genes. We hypothesize that such non-random encoding of the secretion is indirectly selected for in situations when cooperation is favored. Indeed, when evolving populations start from a naive, non-secreting ancestor, the cooperators employed this protective encoding, and more so when the cooperation cost was high. Mutational analysis confirmed that the constrained genetic architecture resulted in cooperation-destroying mutations also having a direct negative fitness effect. Overall, our results highlight the need for considering appropriately detailed and realistic computational systems and generally show the importance of second-order selection pressures and genetic architecture in the study and understanding of the evolution and maintenance of cooperation.

## Results

### Creating a bank of cooperators: General properties

In our modified version of Aevol dedicated to the study of cooperation, the phenotype is divided into two groups of traits: metabolism (biological processes allowing the individual to live and reproduce) and secretion (processes relating to the costly secretion of a diffusible public good molecule). Starting from an ancestor with a single, metabolic gene, we independently evolved 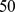 populations for 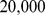 generations. We effectively put cooperation under direct selection by using a particular fitness calculation in which secretion genes were treated the same as the metabolic ones during evolution. At the end of this phase, we chose the fittest individual from each replicate and, by simply reassigning half of the phenotype from metabolism to secretion, obtained 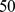 cooperators with high secretion levels. Specifically, their average secretion was 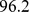
% of the maximal secretion in Aevol, and the standard deviation in secretion was 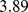
% of the mean. These individuals had generally comparable metabolic and secretion part of their phenotype with on average 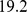 genes in each.

### Cheater invasion dynamics differs between populations

**Figure 1:**
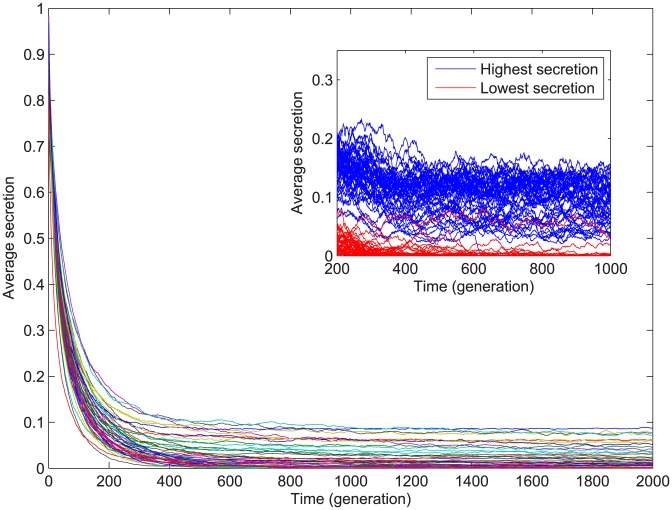
Cheater invasion dynamics in phenotypically similar starting populations. In the main figure each line represents the average secretion, over time, of 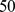 replicate populations started from the same ancestor. For clarity, we did not include the standard error of each group of replicate populations. The insert figure shows all 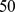 replicate populations for the two most extreme population groups, zoomed in on the time period of interest for our analyses. Specifically, of all the population groups sharing a common ancestor, the blue populations had the highest and red the lowest average secretion between generation 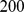 and generation 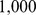. Note that average secretion in the inset represents the average secretion within each population, whilst in the main figure it is the average of the secretion of all the individuals from the 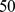 replicate populations that share the same ancestor.

Using the cooperators from previous experiments, we started with 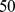 clonal populations that we then let evolve for an additional 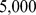 generations with a possibility of secreting at a moderately high cost (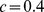, see Materials and Methods for the effect of public good cost and fitness calculation details). Each of these populations was replicated 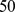 times, for a total of 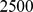 experiments. In all cases the amount of secretion greatly decreased, but not by the same amount or at the same rate (Fig. 1). To quantify these differences, we performed a one-way ANOVA on the average secretion between generation 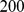 and generation 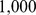, the visually chosen time interval during which cooperation is stabilizing to a new level after a quick and strong decay. We found a highly significant between groups effect (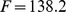, 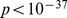), each group consisting of the 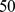 populations that share a common ancestor, confirming that some cooperators are intrinsically more resistant to cheater invasion than others, even though they initially had very similar phenotypes. Moreover, there was no significant correlation between the ancestral cooperation level and the final one (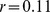, 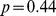), eliminating the possibility of our results being driven by an initial difference in the population cooperation level.

**Figure 2:**
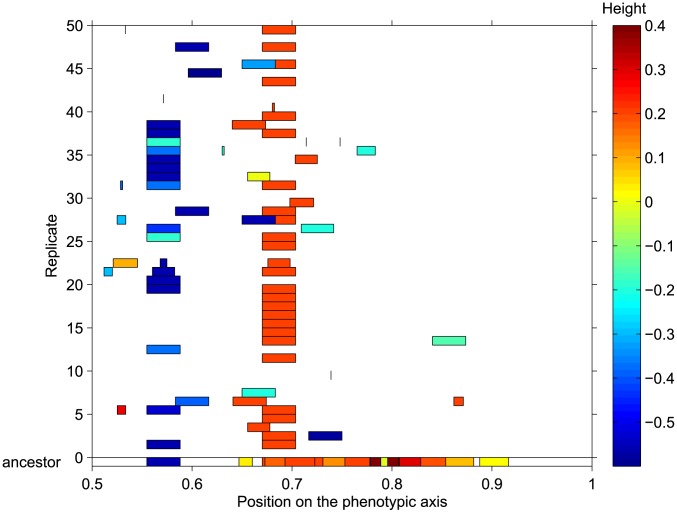
Example of the preferential maintenance of certain secretion genes of a single cooperator from our bank. The bottom row of the graph (ancestor) shows the location on the phenotypic axis of the genes coding for secretion proteins in one cooperator organism from our bank. Above, the secretion genes from the best individual after 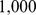 generations of evolution in each of the 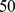 replicate populations descending from this ancestor are shown. Colors represent the height of the proteins encoded by the genes (see Materials and Methods for detailed explanation of protein properties in Aevol).

When visually inspecting the phenotype of a randomly chosen cooperator and its descendants from the previous experiment, we also noticed that it was the same secretion genes that survived cheater invasion between several independent replicates of evolution. One such example, where the phenomenon was especially striking, is presented in Fig. 2. While we did not perform any statistical analysis because of the computational difficulty of tracking every protein for several thousands of generations, this observation motivated further experiments: it supports the idea that our 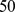 populations are different (in their resistance to cheater invasion) because their secretion genes are somehow different.

### Genetic architecture and resistance to cheater invasion

**Figure 3:**
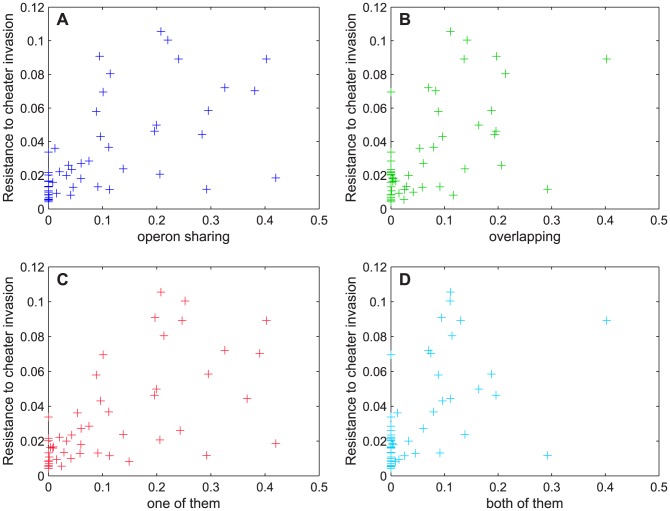
Correlation between the genetic architecture of cooperation genes and the resistance to cheater invasion. Resistance is measured by the amount of secretion surviving cheater invasion. The four types of associations between secretion and metabolic genes shown here are: sharing an operon (A, dark blue), overlapping (B, green), having at least one of the previous two properties (C, red), and having both of them (D, light blue).

To quantify the genetic architecture of 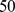 ancestral organisms we measured the percentage of secretion genes that (1) share an operon with at least one metabolic gene, (2) overlap with at least one metabolic gene, (3) satisfy at least one of (1) and (2), or (4) satisfy both (1) and (2) (see Material and Methods for more details). We then compared the genetic architecture measures with the resistance to cheater invasion, expressed as the average remaining secretion between generation 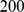 and generation 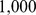, as before (Fig. 3). We found that all four genetic architecture properties strongly correlate with the remaining secretion amount (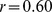 and 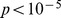 for operon sharing, 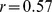 and 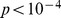 for overlapping, 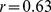 and 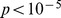 for at least one of them, 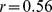 and 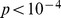 for both of them), supporting our hypothesis that physical linkage between secretion and metabolic genes confers resistance to cheater invasions.

### Mutation effects and resistance to cheater invasion

In order to confirm the effect of genetic architecture on cheater resistance, rather than examining the exact locations and interactions between genes and using them to infer population's evolutionary fate, we directly quantified the effect of mutations on secretion and fitness. We constructed 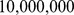 mutants of each of the 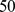 ancestors, and calculated the mutational effect as the percentage of mutations that decrease the amount secreted without decreasing metabolic fitness, weighted by their negative effect on secretion. We found significant correlations between the mutation effect and both the robustness to cheater invasion (calculated as before, 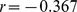, 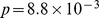) and the genetic architecture (here defined as the percentage of secretion genes sharing an operon or overlapping with at least one metabolic gene, 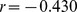, 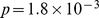). Simply put, the individuals with genetic architecture that groups together metabolism and secretion genes exhibit higher resistance to cheaters, because they are subject to fewer mutations that would convert cooperators into cheaters without any direct fitness loss.

We thus have two measures that predict well the resistance to cheater invasion: genetic architecture and accessibility of mutations. Since the generation range used to quantify cheater resistance was chosen *ad hoc*, we also examined the effect of different ranges on the correlations. Interestingly, we found that genetic architecture is better correlated with cheater resistance when it is measured between generations 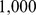 and 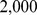 (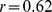 and 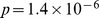) than between generations 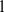 and 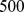 (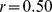 and 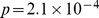). Conversely, mutational effects are better correlated with cheater resistance measured in the early (generations 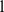 to 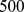, 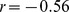 and 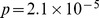) than late interval (generation 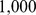 to 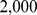, 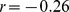 and 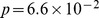). Overall, both genetic architecture and mutation effects are good predictors of how easily cooperators may be invaded by cheaters, but genetic architecture is better at predicting long-term effects, while mutational effects are more strongly correlated with short-term ones. Mutations may affect long-term maintenance of cooperation in many ways and genetic architecture captures but one of them. As we elaborate in the Discussion section below, these results indicate that while all mutational constraints play a role, it is the overlap and operon ones that have the strongest long-term evolutionary consequences.

### Preferential maintenance of secretion genes based on genetic architecture

**Figure 4:**
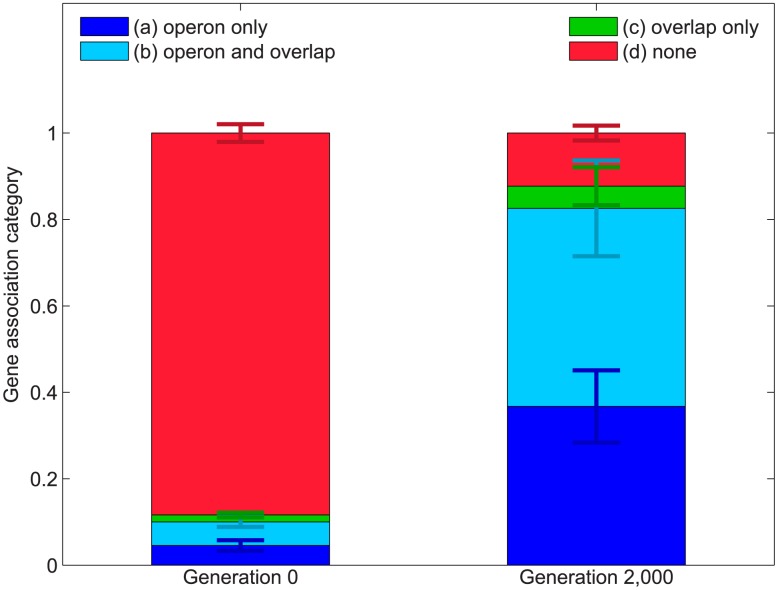
Secretion genes at generation 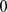 and at generation 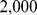 partitioned between four categories: (a) sharing a promoter (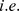 being on the same operon) without overlapping with a metabolic gene (dark blue), (b) overlapping and sharing an operon with a metabolic gene (light blue), (c) overlapping without sharing an operon with a metabolic gene (green), (d) neither sharing an operon nor overlapping with a metabolic gene (red). Error bars represent one standard error of the mean (fifty original cooperators). The color of the error bars corresponds to the genetic architecture category which they relate to.

We tested the importance of gene overlap and operon sharing in maintenance of cooperation by examining the extent of genomic connections between secretion and metabolism before and after the increase in secretion cost and the accompanying decrease in cooperation. After 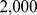 generations of evolution at a higher cost, the secretion genes still present are over 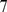 times more likely to overlap or share an operon with metabolic genes than the secretion genes a the start of the experiment (Fig. 4), with the difference being highly significant (Welch's t test, 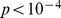). The proportion of all four categories of association between metabolic and secretion genes (share an operon, overlap, do at least one of them, do both) has increased, and all increases were significant (Welch's t test, 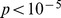 for operon sharing, 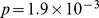 for overlapping, 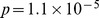 for doing at least one of them, 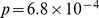 for doing both). Note that these categories do not exactly correspond to the partitioning done on Fig. 4 (see Material and Methods for detailed explanation), but capture the same general properties of genetic architecture.

### Evolution of genetic links between metabolic and cooperation genes

**Figure 5:**
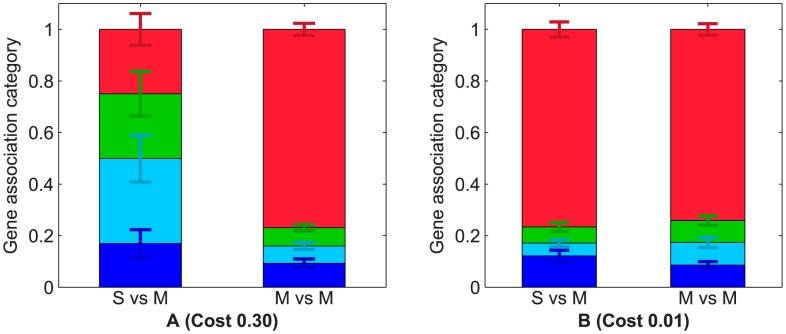
Genetic architecture associations after 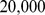 generation of *de novo* evolution of cooperation, at (A) high, c = 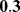 and (B) low, c = 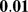 secretion cost. In both cases, we quantified the presence of operons or overlaps between secretion genes and metabolic genes (S vs M) as well as between metabolic genes and other metabolic genes (M vs M). Genes were partitioned in four categories, labeled as in the Fig. 4: sharing a promoter (an operon) without overlapping with a metabolic gene (dark blue), overlapping and sharing an operon with a metabolic gene (light blue), overlapping without sharing an operon with a metabolic gene (green), not sharing an operon nor overlapping with any metabolic gene (red). Error bars represent plus and minus one standard error of the mean for the fifty replicate populations and their color corresponds to the genetic architecture category they relate to.

In the previous experiments we worked with already evolved cooperators, measured their resistance to cheater invasion and genetic architecture. We now turn to *de novo* evolution of cooperation, in order to show that gene overlap and operon sharing will evolve, via indirect selection pressures, in conditions moderately favorable for cooperation. Naive ancestors evolved for 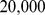 generations with cooperation cost of 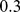. In the final populations, individuals on average had 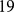 metabolic genes and 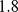 secretion genes. While the number of secretion genes is low, by pooling data of all 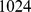 individuals from each population we obtained a large number of genes that we could analyze. We compare the shared operons and gene overlap for metabolic and secretion genes with the same measures applied only within metabolic genes, as a control. The genetic architecture links between metabolic and secretion genes are on average 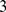 times stronger than within metabolic genes alone, the difference being highly significant (Fig. 5a, comparing the sum of the three bottom categories – dark blue, light blue and green –, Welch's t test, 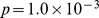). The proportion of all four genetic architecture categories differed between the two gene groupings, and all differences were significant (Welch's t test, 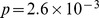 for operon sharing, 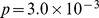 for overlapping, 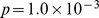 for at least one of them, 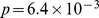 for both). We repeated the analysis for 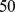 more populations that evolved under a lower secretion cost (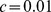) and we observed no difference in genetic association between metabolic and secretion genes compared to associations with metabolic genes alone (Fig. 5b, Welch's t test, 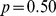). Comparison of the two sets of experiments performed at different secretion costs shows that the preferential association between secretion and metabolic genes evolves only when the cost of cooperation is relatively high.

## Discussion

Our research was motivated by two simple observations: (1) phenotypically near-identical populations have different evolutionary fates (Fig. 1), and (2) for a given cooperator, the secretion genes that survive cheater invasion seem to be always the same between several replicates (Fig. 2); and by two straightforward questions: what were the differences between theses populations, and what were the differences between these genes? In our initial experiments we found that when populations cooperating at near-maximal level suddenly faced a direct, high cost of public good secretion, the cheaters were quick to invade. However, the invasion dynamic was qualitatively different across populations, with some ancestors predictably evolving into populations without any cooperation, while others kept low but non-zero levels of secretion (Fig. 1).

We propose that these diverging evolutionary fates for otherwise phenotypically similar populations are due to differences in the ancestral genetic architecture of cooperative traits. In a previous paper we showed that the accessibility of mutations impacting secretion may lead to different future secretion dynamics for phenotypically similar individuals [25]. Here we show that in the case of cooperation decay, beyond the simple effect triggered by the accessibility of mutations impacting secretion, the way secretion genes “share” the genome with metabolic genes also has an effect on the selection of these mutations. More precisely, we suggest that secretion genes that are physically connected with a metabolic gene, for example belonging to the same operon, or physically overlapping, are more robust to cheater invasion: a deleterious mutation in one of these genes is more likely to also deleteriously affect a metabolic gene, and thus is less likely to be selected for. Similar mechanisms, coupling cooperative traits with metabolic, individualistic traits have been described before [16], [17], but instead of relying on gene coregulation or genes with multiple effect, we report a more basic genetic mechanism of entanglement for genes with singular effects. Additionally, these studies remain two isolated data-points and are thus not enough to show the existence of second order selection pressures leading to such architecture. Bio-informatics methods could provide much more information to support or deny this hypothesis, however there have been very few relevant studies of the genes involved in cooperative behavior, the primary reason being the difficulty of identifying such genes. There is one notable exception [26], where the authors use the prediction of cellular localization: outer membrane and excreted proteins are more likely to be related to cooperative traits than cytoplasmic, inner membrane and periplasmic proteins. Their main result is about the role of horizontal transfer in the evolution of cooperation, however they also show that genes coding for outer membrane and excreted proteins are more likely than others to be “genome neighbors” of addictive systems (e.g. toxin-antitoxin). As the experimental data is suggestive but overall still insufficient, the use of an appropriate individual based model dedicated to the study of evolutionary processes and selection pressures acting on the genome, such as Aevol, is a way to fill the void. Of course, each model has biases and limitations, however, the strong point of Aevol is that we implement only simple, easy to understand, small-scale rules inspired by bacterial genomics, and all other properties and processes are emergent. For example, in Aevol there is no parameter like “probability that two neighboring genes overlap”. Thus, the outcomes we describe here are not directly driven by the model and are not something we necessarily expected to evolve.

Using Aevol we were able to directly test our hypothesis about the effects of genetic architecture on the evolution and maintenance of cooperation we generated and analyzed a total of 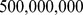 mutant organisms. About half of the mutants are phenotypically perfectly similar to the original individual (no mutations or only neutral mutations happened). The calculated probability of having no mutations for typical organism with genome length of 10,000 is 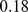. The calculated probability of having exactly one mutation is 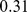, and the one of having strictly more than one mutation is 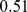. We emphasize that a large part of the analyzed mutations are neutral but of course focus on the ones that change organisms fitness and secretion. We recorded the effect of all mutations, specifically searching among “cheating” mutations, the ones that would decrease the amount of secretion, for mutations that do not simultaneously decrease metabolic fitness, and may thus be selected for, or at least not immediately purged by selection. We find that the proportion of these mutations, weighted by their negative effect on secretion, directly and significantly correlates with the population's vulnerability to cheater invasion, which supports our hypothesis. Interestingly, when we measured the remaining cooperation later in time it correlated more strongly with amount of genetic architecture linkage between secretion and metabolism than the measured effect of the introduced mutations. The higher durability of genetic architecture constraints, compared to immediate mutation effects, may be due to the higher durability of the genetic architecture itself. As we saw from the comparison between different gene association categories at generation 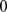 and generation 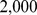, it is exactly the secretion genes that do overlap or share an operon with metabolic genes that may be preserved (Fig. 4). On the other hand, our mutational analysis explored only a small, nearby portion of the immense fitness landscape. As organisms evolve, and move around that landscape, the particular mutants we constructed and analyzed may become less accessible via mutations and thus also less relevant for the evolutionary dynamics. Finally, the enrichment of genotypically connected metabolic and secretion genes among all secretion genes present in the individuals strongly suggests that this type or architecture may generally be created and maintained via indirect selection in cooperative systems.

We should note that in our first set of experiments we used individuals that evolved under an altered fitness calculation regime that enabled us to directly select for future cooperators of similar phenotype but different genotype. We could have as well designed these individuals by hand, directly writing the zeros and ones in their genomes. However, this would have likely generated fragile and generally poorly adapted individuals, as evolution is typically better in organism design than us humans. The change between the alternative fitness calculation and the regular one may appear somewhat artificial or arbitrary, but it can also be seen as a transition from producing a private, non-secreted good, to a public, secreted one. Still, while these results show the effect that architecture may have on cooperation, they do not by themselves prove the existence of second order selection pressure that would be sufficient to create operon and overlap type constraints during the evolution and maintenance of cooperation. We thus turn to our second set of experiments, in which secretion evolves *de novo*, without ever being directly selected for.

The results of this second set of evolution experiments strongly support the hypothesis that when costly cooperation does evolve and persist, there is a selection pressure grouping secretion genes with metabolic genes to protect them from removal. Such selection pressure is necessarily indirect, since cooperation via public good secretion does not directly increase the fitness of the cooperating organisms. Additionally, it would not prevent any “cheaters” from appearing but would reduce their likelihood of having a greater fitness than cooperators and spreading, making overall conditions more favorable for cooperation by reducing the effective mutation rate for switching from being a cooperator to being a potentially invading cheater. Previous work has already established this “mutation rate” (in a binary, game theory vocabulary) is one of the very important parameters controlling the dynamics of cooperation in spatially structured populations [27], [28]. Here we extend these results by showing a specific genetic mechanisms that would allow evolution to modulate the rate of switching between potentially invading cheaters and cooperators. The total rate of production of cheaters may not be different between populations, but because of the genetic entanglement of cooperation and metabolism, a large proportion of cheaters is unable to invade and thus such cheater mutants are evolutionary dead-ends.

The role of constraints introduced by second-order selection, such as the one we exemplified here, in assuring the best long-term outcomes has been proposed before in a more general and abstract context [29]. Specifically, our results may provide a set of new potential explanations for the evolution of operons and overlaps, important building blocks of life. While in the past operon and overlap existence has been linked to co-regulation and co-transfer of genes working together and belonging to the same sets of biological processes [19], here we highlight their role as an evolutionary constraint. Specifically, operons and overlaps may protect genes that are at risk of removal because of a short-term cost and in spite of the long-term benefit they may provide. Of course, the particular combination of short-term cost and long-term benefit is not unique to cooperation and it underlies other biological processes, most notably sex and recombination, which also continue to be intensely studied [30]. In terms of cooperation itself, genetic architecture constraints may be highly relevant in understanding the much studied siderophore-mediated cooperation in *P. aeruginosa*, where cooperative as well as essential metabolic traits are under the control of a quorum sensing mechanism [17]. However, our idea also has large implications outside of the field of cooperation: going beyond explaining evolutionary outcomes, the genetic architecture coupling mechanism we describe here could be actively used to prevent mutations from removing of genes introduced into bio-engineering organisms, one of the major problems in the field of synthetic biology [31].

### Conclusion

The study of the evolution and maintenance of cooperation is rich in theories, majority of which rely on higher level properties of individuals, such as relatedness, fitness, or group structure. Our experiments investigate basic, genome-level properties and show that the presence of genetic associations between metabolism and secretion genes aids the maintenance of cooperation across thousands of generations. Operon sharing and gene overlap are selected for when cooperation is costly and directly change populations' evolutionary fate. Second order selection is known to play a major role in the rapid evolution of microbial populations [32] and here we contribute to understanding the specific and much studied case of cooperation via public good secretion. We used an *in silico* experimental platform, Aevol, which has enabled us to collect and analyze genetic architecture and evolutionary dynamics data in detail previously unattainable with either mathematical or experimental systems. The role of second-order selection and genetic constraints in evolution will undoubtedly continue to motivate experimental and theoretical research but in our case it also has the potential to inform bio-engineering and synthetic biology applications.

## Materials and Methods

### Aevol digital evolution system

In this study we use the Aevol platform, an individual-based model of evolution, especially well suited for the study of selection pressures on genomic architecture [21], [22], [25], [33]. It is free and open-source software and is downloadable from http://www.aevol.fr/download. The specific version of the platform we used in this study, including analysis routines, parameter files, other minor modifications, is available on request. Aevol has already been used in several peer-reviewed publications including some that studied cooperation, so we invite the reader to refer to [34] for more information on how cooperation has been implemented and for characterization of the related parameters, and to [22], [35] for more general details about the original version of Aevol that did not incorporate cooperation.

In Aevol, the individuals are living on a toroidal, two-dimensional square grid, with each location being occupied by exactly one individual. In our experiments the grid contains 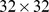 positions, for a total of 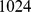 individuals. Selection and reproduction are performed locally in a synchronous way: at each generation, for each position in the grid, we compete the nine individuals in the neighborhood to determine which one's descendant in going to occupy this position in the next generation.

The phenotype of an individual is represented by a two-dimensional curve describing the level of performance for each point of a continuous set of abstract biological processes. This is a very general way of encoding a phenotype without any restriction on the type of biological processes that can be represented. The genotype is a string of zeros and ones, which is transcribed and translated according to a bacterial genomics-inspired process: promoters and terminators are identified to allow transcription, then the transcribed sequences are searched for ribosomal binding site and start codon, followed by what will be a gene and then by an in-frame stop codon, to allow translation.

**Figure 6:**
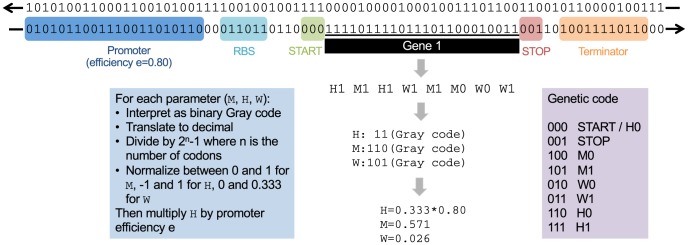
Aevol genetic code. Here we use an example of a functioning gene from an Aevol individual to explain the transcription and the translation processes. The gene is flanked by a promoter and terminator regions and preceded by a ribosome binding site (RBS). The codons for mean position, the width, and the height of the protein are identified, transformed into Gray code using the Genetic code table (box on the right), and finally scaled and normalized, as we summarize in the box on the left and describe in more detail in the Methods. Note that a gene with re-shuffled codons, for example H1 H1 M1 M1 M0 W1 W0 W1, would encode exactly the same protein. START codon may occasionally be found inside a gene, in which case it is interpreted as H0. The promoter differs from the consensus sequence by 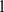 base out of the maximal 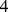 differences allowed, giving it a 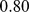 efficiency.

**Figure 7:**
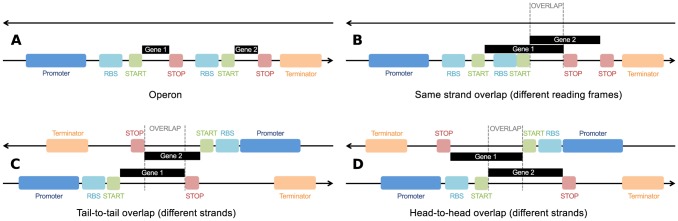
Examples of constrained genetic architecture in Aevol. (A) As is often the case in natural systems, here the two digital genes belong to the same operon. They share a promoter and a terminator sequence and are thus being expressed at the same level. These hypothetical genes would belong to the “operon only” category from Fig. 4 and Fig. 5. (B) These two genes also share the same operon but additionally their sequences overlap, putting them in the “operon and overlap” category. In this case, the genes are in different reading frames and do not share a STOP codon, although such configuration is also possible. As the black gene boxes indicate, the left STOP codon corresponds to the left START codon, while the right STOP codon corresponds to the right START codon. (C, D) Two examples of genes from the “overlap only” category which are encoded on different strands. This is not an exhaustive list of possible genetic constraints, as a gene may, for example, share an operon with a gene on the same strand while simultaneously overlapping with a gene on an opposite strand.

Our genetic code is an abstract mathematical function transforming the gene, i.e. the binary sequence between the start and stop codons, into three numbers, interpreted as a triangle on the axis of biological processes. These three numbers are 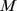 (mean position of the triangle on the phenotypic axis), 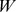 (half-width of the triangle), and 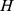 (height of the triangle). Base-pairs are read three by three, and our amino-acid space has eight symbols: START and STOP, M0 and M1 which are used to specify 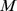, W0 and W1 which are used to specify 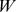, H0 and H1 which are used to specify 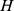 (Fig. 6). Each of these eight amino-acids is assigned to exactly one of the eight (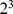) possible triplets. There is no redundancy in the codon–amino-acid mapping, however there is still a large redundancy in the gene–protein mapping because codons inside genes can be reordered without impacting phenotype. Specifically, what matters is the order of codons specifying the same triangle property (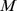, 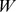, or 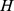), while the relative order of codons for different properties can be altered freely. Once a coding sequence has been detected using the rules explained above and transformed into an amino-acid sequence, we extract from there three binary words (for 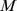, 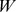 and 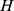) according to the following process: amino acid X0 adds a 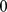 to the binary word of 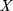 and X1 adds a 1 to the binary word of 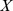, where X is any of M, W, or H. We obtain an integer value for each of the three binary words by interpreting them using Gray code. Gray code is an alternative binary encoding in which two successive integers are encoded by binary numbers differing in only one digit. The integer values are then normalized by 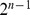 where 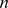 is the number of codons used, and scaled to a [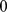, 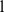] interval for 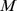, [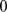, 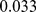] for 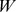, and [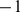, 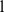] for 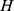. Finally the 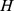 value is multiplied by the transcription efficiency – a property of the promoter explained below. The mean position specifies the primary trait the protein affects, and as it is a real number, it allows for an infinite number of different traits. The height specifies protein's performance level for the primary trait, while the width determines all the traits a protein affects. Individual's phenotype is computed by summing up all the triangles encoded in its genome. There is no genetic regulation via transcription factors in this version of Aevol, however there are protein-protein interactions (two proteins contributing to the same biological processes) and transcription efficiency is regulated by the strength of the promoter (defined as the distance to a consensus sequence). As in natural systems such as bacteria or phages, this genomics allows two genes to cluster on the same mRNA (operon) or to physically use the same DNA basis in different reading frames or different senses (overlap). Examples of these different configurations are represented on Fig. 7.

The environment is represented by a two-dimensional curve indicating what is, for every possible biological process, the optimal level of performance in the given environment. The fitness of an individual is a decreasing function of the distance between the individual's phenotype and the optimal phenotype. Individuals are locally selected according to their rank in the neighborhood, with a probability of reproduction exponentially decreasing with the rank. The chosen individual will undergo reproduction with mutations (insertion, deletion or substitution of a small number of basis and duplication, inversion, translocation or deletion of a larger portion of the genome). The rates of different mutation types are parameters of the model and have been set to 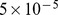 per basis for small mutations, and 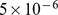 for large mutations. Ancestral genome is 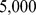 bases long and contains a single gene, while the typical genome length after several thousands of generations of evolution is around 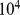 basis. Aevol is a stochastic simulation, the variability coming from the randomness of mutations and the probabilistic selection. One of the parameter is the random seed used to initialize the random number generator. We can replicate an experiment by running it several times with the same exact parameters, but different random seeds.

In our experiments, we distinguish two categories of biological processes: the “metabolic” ones (all traits positioned before 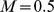 on the axis of the biological processes), that allow an individual performing them to live and reproduce, and the “secretion” ones (position after 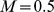 on the axis), that determine the level of the production of the public good. We note that under our setup, while genes are generally pleiotropic, simultaneously influencing multiple traits, it is not possible for a gene to affect both metabolic and secretion traits. The public good is costly to secrete, but diffuses in the environment and is beneficial to every individual that comes in contact with it. The cost for the production of one unit of public good varies in our experiments, but is always equal to the cost coefficient (parameter we set) multiplied by the amount of the public good produced. The fitness of an individual is given by this equation:
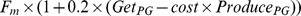
Where 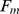 is metabolic fitness (calculated as explained before but only considering the left part of the axis), 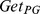 is the amount of public good present in the environment at the location the individual inhabits, 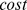 is the per-unit cost of the public good production, and 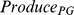 is the amount of pubic good produced by the individual (computed similarly to metabolic fitness but considering the right part of the axis). 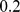 is a constant chosen based on previous experiments [25].

The diffusion parameter is 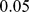 per generation, meaning that five percent of the public good present at one position will diffuse in each of the eight neighboring positions during one generation. The degradation rate is set to 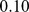 per generation, meaning that ten percent of the public good at each location will degrade during one generation. This degradation can be thought of as replacing any explicit consumption of the public good, but also as specifying the public good durability. Overall, in all our experiments, 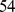
% of the public good present at generation 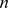 at some position will remain at this position at generation 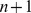. In the Supporting Text S1, we experimentally show that the secretion mechanism implemented in Aevol, as described above, leads to the usual cooperation dilemma.

### Evolving a bank of cooperators

To evolve a large number of strong cooperators, we assigned biological processes that were usually in the secretion part of the phenotype to the metabolic part of the phenotype, allowing a strong direct selection on them. After 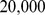 generations of evolution under these conditions, the whole phenotype of the individuals closely matches the target phenotype. Thus, when picking the best individual and re-assigning half of the trait axis back to secretion, we get a “near-perfect” cooperator, one that secretes close to the maximal possible amount of the public good. Evolving cooperators in this way makes secretion genes evolve in the same way as the metabolic ones, to a high level, increasing the potential signal in further experiments. We repeated this experiment 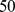 times, extracted the fittest individual from each population, and obtained a bank of 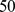 independently evolved cooperators.

### Analysis of the genomic architecture

For each of the 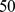 cooperators we evolved in the first set of experiments, we analyzed the architecture of all its secretion genes and classified them in four different categories: (1) genes that share an operon with at least one metabolic gene without overlapping with a metabolic gene, (2) genes that overlap with at least one metabolic gene without sharing an operon with a metabolic gene (this is possible because our digital DNA is double stranded and thus allows for two reading senses, in addition to three reading frames for each sense), (3) genes that overlap with at least one metabolic gene and share an operon with at least one metabolic gene (not necessarily the same one), and (4) genes that share neither operon nor overlap with a metabolic gene. There are multiple ways one could classify the different genes, for example, by distinguishing the number of metabolic genes a secretion gene overlaps or shares an operon with. The four categories we chose have the benefit of intuitive simplicity in addition to including all secretion genes in exactly one category, and we have used them in Fig. 4 and Fig. 5. The number of genes in each category is always shown as a percentage of all the secretion genes and standardized by the genes' phenotypic area. Here, the phenotypic area refers to the area of the protein (triangle) the gene encodes for, and allows us to give more weight to the genes that have a strong impact on secretion as well as enable comparison between replicate experiments that may have different secretion levels.

However, when performing the statistical analyses to determine the correlation between the presence of overlap and the resistance to cheater invasion, it does not makes sense to, for example, exclude the secretion genes that also share an operon (in addition to overlapping) with a metabolic gene. So we use slightly different, larger, categories for secretion genes: share an operon with at least one metabolic gene (which is exactly the addition of categories 1 and 3 of our previously explained partitioning), overlap with at least one metabolic gene (addition of categories 2 and 3), do at least one of them (addition of categories 1, 2 and 3), do both of them (same than category 3), do none of them (same than category 4). The difference is that these categories are no longer exclusive: one gene can be in more than one of the new categories at the same time. The genes are standardized by their phenotypic area, as before. These regrouped categories are used in the statistical analyses throughout the paper, and can easily be visually inferred from the categories of the bar graphs in Fig. 4 and Fig. 5.

### *De novo* evolution of cooperation

In these experiments, each population starts from a randomly constructed organism with a 5,000 base pair genome. As random sequences of 0's and 1's are generated, they are screened for the presence of open reading frames with genes. Thousands of sequences are tested and the first one that has exactly one metabolic gene with a positive effect on fitness is selected. This genome is then cloned to fill the population grid and form the starting population. Reason for starting with a single, valid gene rather than an organism with effectively empty genome is that in both cases all the genes except the first one have a very high probability of evolving from duplication followed by divergence of one already existing gene. Indeed, promoters and ribosome binding sites are hard to evolve from scratch. Starting from purely random sequence would only greatly slow down the evolution process (genomes could evolve for thousands of generation before the first gene appears [22]) without qualitatively changing the understanding of the evolutionary process in our system. After 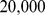 generations of *de novo* evolution, we pooled the proteins from all the individuals in each replicate to obtain a measure of average genetic architecture within a population. As before, rather than using just a protein count, we standardized the contribution of each protein by its phenotypic area.

## Supporting Information

Figure S1
**Individuals are tempted to stop cooperating.** For each of the 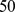 populations, we plot the average fitness increase an individual would experience if it would individually stop cooperating, *i.e.* the temptation to defect, against the average amount secreted by an individual in the population. Except in the populations where no cooperation has evolved (red points), the temptation is always greater than zero.(PDF)

Figure S2
**Groups of cooperators do better than groups of defectors.** For each of the 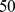 populations, we plot the benefit of cooperation, *i.e.* the average fitness drop individuals would experience if cooperation was disabled, against the average amount secreted by an individual in the population. Except in the populations where no cooperation has evolved (red points), the benefit is always greater than zero.(PDF)

Figusre S3
**Individuals that cooperate more are the ones that benefit more from secretion.** For each of the 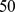 populations, we plot the correlation between how much individuals secrete, and how much they benefit from secretion (*i.e.* the average fitness drop individuals would experience if cooperation was disabled), against the average amount secreted by an individual in the population. Except in the populations where no cooperation has evolved (red points), the correlation is significant and positive for all but 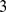 populations.(PDF)

Text S1 **Secretion of a public good in Aevol satisfies the two usual requirements for a social dilemma.** The fitness of an individual would always increase if it would stop cooperating (Supporting Figure S1), and in a group of cooperators the individuals have a higher fitness than in a group of cheater (Supporting Figure S2).
(PDF)
