# Supplementary figures and images for: Genetic Architecture Promotes the Evolution and Maintenance of Cooperation
Source: PLoS Comput Biol. 2013 Nov 21;9(11):e1003339. doi: 10.1371/journal.pcbi.1003339 (PMC3836702; doi:10.1371/journal.pcbi.1003339)

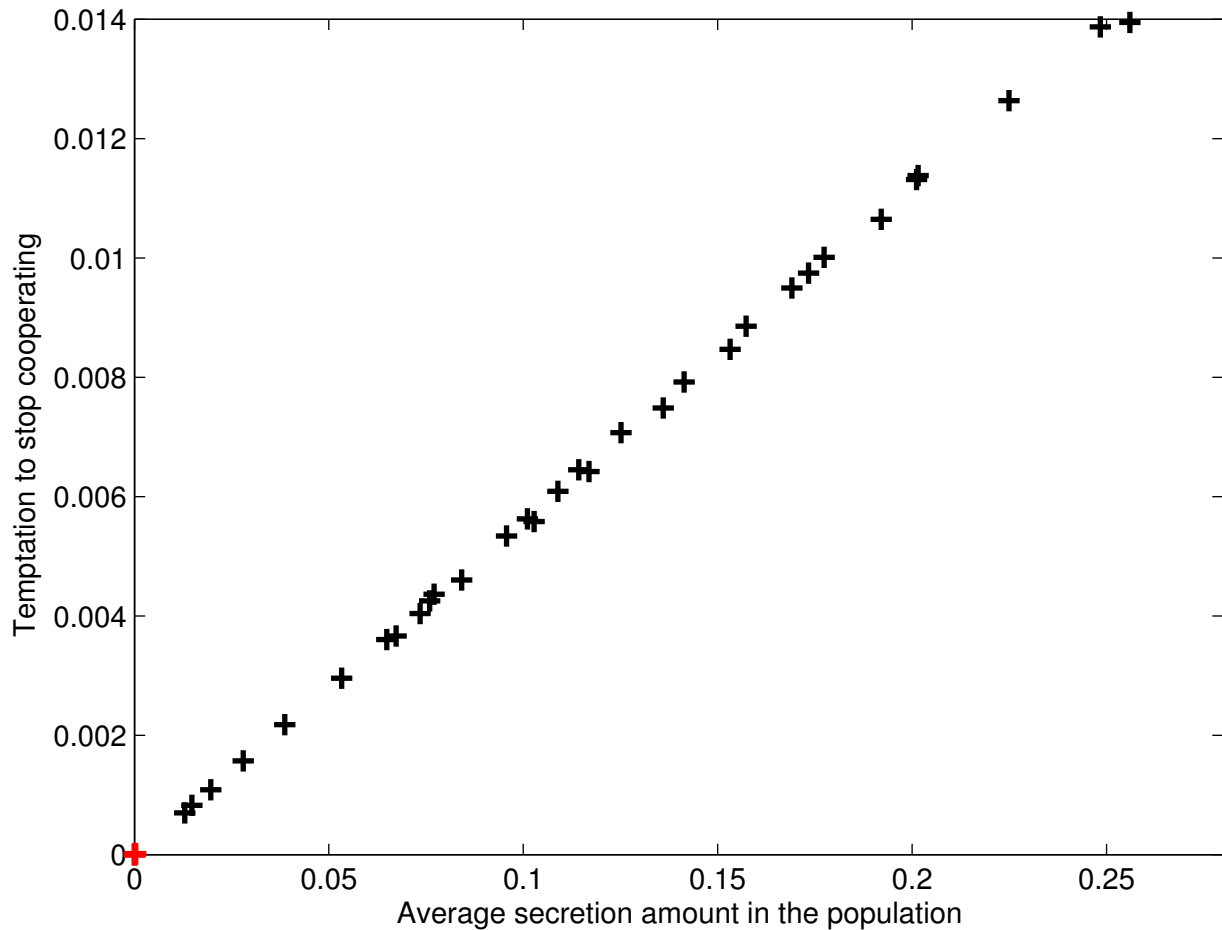

Supplement: Figure S1 — Individuals are tempted to stop cooperating. For each of the populations, we plot the average fitness increase an individual would experience if it would individually stop cooperating, i.e. the temptation to defect, against the average amount secreted by an individual in the population. Except in the populations where no cooperation has evolved (red points), the temptation is always greater than zero. (PDF) [file pcbi.1003339.s001.pdf]

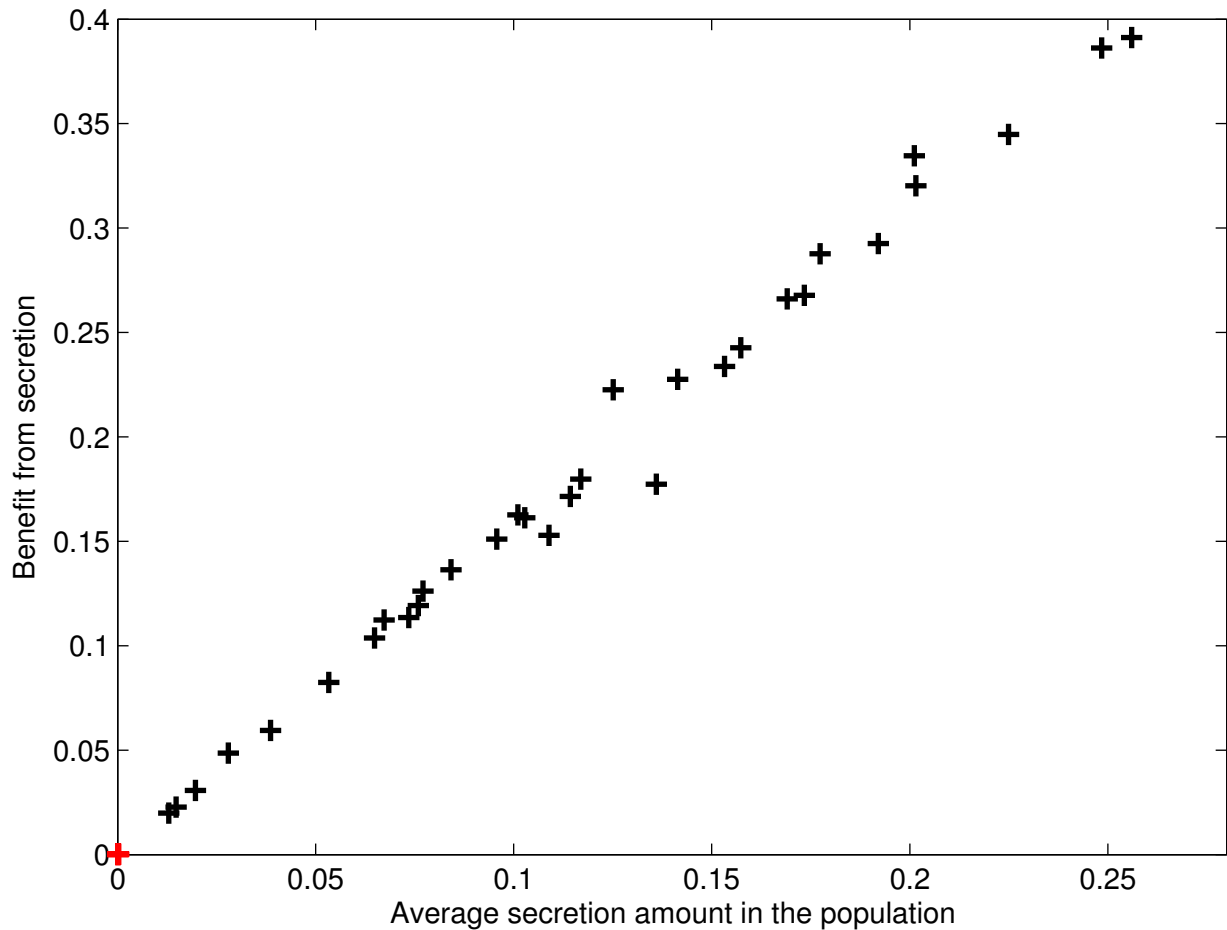

Supplement: Figure S2 — Groups of cooperators do better than groups of defectors. For each of the populations, we plot the benefit of cooperation, i.e. the average fitness drop individuals would experience if cooperation was disabled, against the average amount secreted by an individual in the population. Except in the populations where no cooperation has evolved (red points), the benefit is always greater than zero. (PDF) [file pcbi.1003339.s002.pdf]

Correlation between benefit from secretion  
and amount secreted

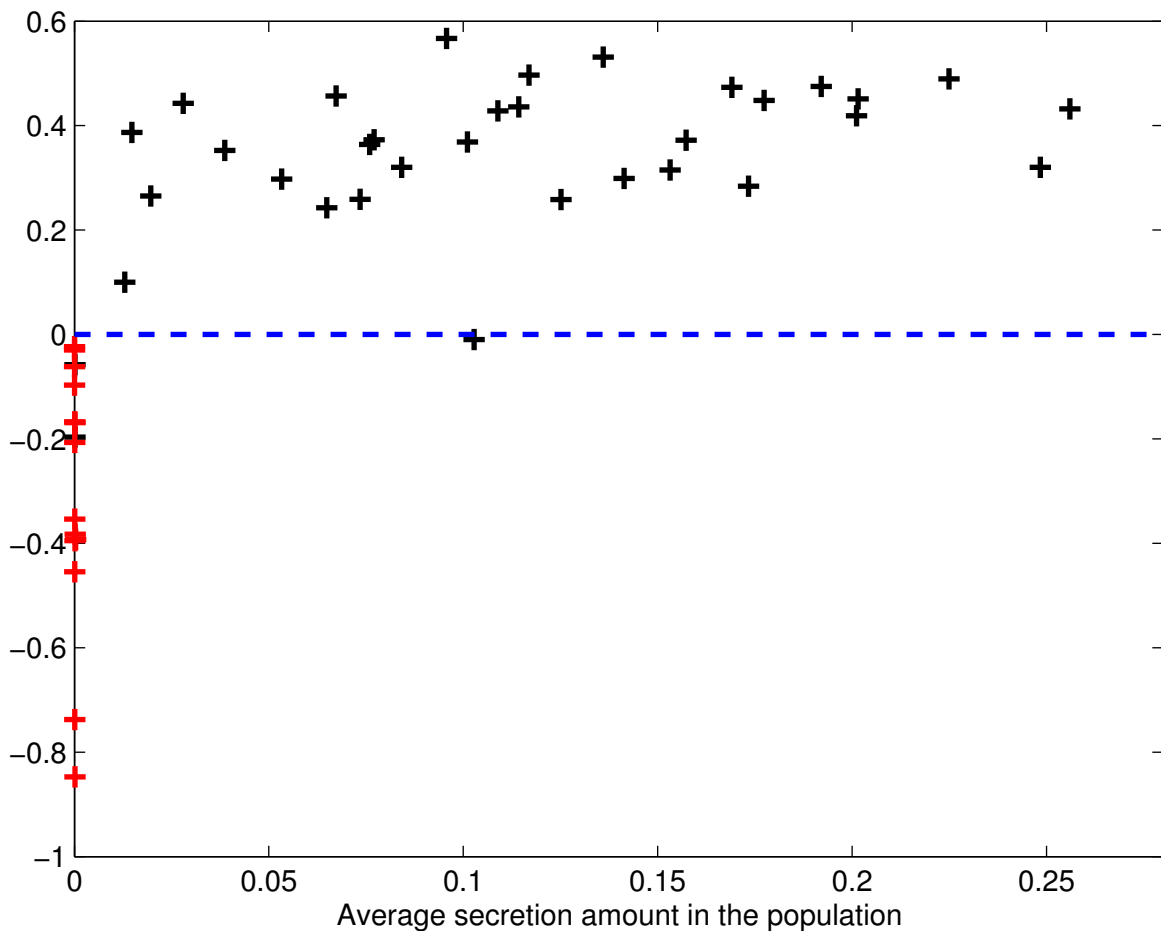

Supplement: Figusre S3 — Individuals that cooperate more are the ones that benefit more from secretion. For each of the populations, we plot the correlation between how much individuals secrete, and how much they benefit from secretion (i.e. the average fitness drop individuals would experience if cooperation was disabled), against the average amount secreted by an individual in the population. Except in the populations where no cooperation has evolved (red points), the correlation is significant and positive for all but populations. (PDF) [file pcbi.1003339.s003.pdf]
