# Supplementary material for: Genetic Architecture Promotes the Evolution and Maintenance of Cooperation
Source: PLoS Comput Biol. 2013 Nov 21;9(11):e1003339. doi: 10.1371/journal.pcbi.1003339 (PMC3836702; doi:10.1371/journal.pcbi.1003339)
Supplement: Text S1 — Secretion of a public good in Aevol satisfies the two usual requirements for a social dilemma. The fitness of an individual would always increase if it would stop cooperating (Supporting Figure S1), and in a group of cooperators the individuals have a higher fitness than in a group of cheater (Supporting Figure S2). (PDF) [file pcbi.1003339.s004.pdf]

Genetic Architecture Promotes the Evolution and Maintenance of Cooperation
Supporting Text S1

Antoine Frénoy*, François Taddei and Dusan Misevic
INSERM U1001, Université Paris Descartes, Sorbonne Paris Cité
* E-mail: frenoy@gmx.com

Cooperation in Aevol

Simulations have historically played a major role in increasing our understanding of the evolution and maintenance of cooperation in nature. Much of the research has followed a game theory paradigm, where at each generation, each individual is playing a prisoner's dilemma-like game with each of their neighbors. In such a setup it is rather easy to demonstrate that the exhibited behavior is indeed cooperation, as it just depends on the specific game played and parameters used. However, in our research platform, Aevol, cooperation is the production of a secreted public good that is explicitly subject to diffusion and degradation dynamics. Such implementation of cooperation makes things a little bit more complicated: the interactions between individuals can not be described using a pure game theory framework, because fitness/payoff of a focal individual depends not only on its own and its neighbors' behavior/strategy, but also on the history of the public good production in a large, neighboring portion of the space population inhabits.

In order to support our main finding, that genetic architecture is selected because it allows the maintenance of cooperation, we would first need to demonstrate that the secretion we see in Aevol is not just a deleterious/neutral bi-product of generic drift or constrained genetic architecture, but a process that is indeed favorable to the individuals performing it. We can briefly summarize the usual requirements for cooperation dynamics as follows: (1) for any individual, it pays more to defect than to cooperate, and (2) in a group of cooperators, the individuals do better than in a group of cheaters. In order to show this, we performed additional analysis of the 50 replicate populations evolved in our second experiment (*de novo* evolution of cooperation, cost of cooperation $c = 0.3$), at generation $20,000$. Besides recording the average amount of public good secreted by each individual and their fitness w, we also calculated two other, hypothetical fitness values: w_{no_sec}, a fitness individual would have if it were not secreting but could still benefit from the public good present in the environment, and w_{no_coop}, a fitness individual would have if it would experience neither the cost nor the benefit of cooperation. This allows us to compute both the cost of cooperation, w_{no_sec} - w, as well as the benefit gained by cooperating, w - w_{no_coop}. Finally, rather than only looking at average values across the population, for all individuals in a population we also calculate the correlation between the amount secreted and the benefit of cooperation.

Using the empirical calculations of cost and benefit of cooperation, we can directly show that in all our experiments, there indeed is a temptation to stop cooperating, as on average, in each of our 50 populations the fitness of an individual would always increase if it would stop secreting (requirement (1) above, Supporting Figure S1). We also show that in the group of cooperators, individuals do better than in the group of cheaters, as the average fitness in the population would decrease if cooperation was completely disabled (requirement (2) above, Supporting Figure S2). Finally, we examined the 34 populations in which the average amount of secretion was significantly greater than zero (Wilcoxon signed rank test, with $p < 0.05$),

and in all but three we find that there is a positive significant correlation between the amount an individual secretes and the benefit he gets from cooperation (measured as the Pearson's correlation coefficient r, with p-value $p < 0.05$), Supporting Figure S3. This reinforces the point that cooperation process in Aevol satisfies the requirement (2) and is a direct consequence of the spatial structure (viscosity) of the population, allowing non-random assortment of individuals.

Based on the analysis above, we can rule out the possibility that secretion is detrimental for the population but is maintained due to constrained genetic architecture or genetic drift. Instead, we conclude that the secretion behavior that evolves in our experiments satisfies the usual definition of cooperation and causes the standard dilemma (interest of the individual versus interest of the group), and that it may be selected for due to spatial structure. This provides a solid basis for Aevol to be used as an digital platform for the study of cooperation in the current, as well as previous and future studies.
